# Bioaccumulation of heavy metals in some commercially important fishes from a tropical river estuary suggests higher potential health risk in children than adults

**DOI:** 10.1371/journal.pone.0219336

**Published:** 2019-10-17

**Authors:** A. S. Shafiuddin Ahmed, Sharmin Sultana, Ahasan Habib, Hadayet Ullah, Najiah Musa, M. Belal Hossain, Md. Mahfujur Rahman, Md. Shafiqul Islam Sarker

**Affiliations:** 1 Department of Fisheries and Marine Science, Noakhali Science and Technology University, Noakhali, Bangladesh; 2 Department of Chemistry, National University, Gazipur, Bangladesh; 3 Faculty of Fisheries and Food Science, Universiti Malaysia Terengganu, Kuala Nerus, Terengganu, Malaysia; 4 Southern Seas Ecology Laboratories, School of Biological Sciences, The University of Adelaide, Adelaide, South Australia, SA, Australia; 5 Department of Statistics, Cumilla University, Cumilla, Bangladesh; 6 Forensic Science Laboratory, Rapid Action Battalions Headquarters, Dhaka, Bangladesh; Chinese Research Academy of Environmental Sciences, CHINA

## Abstract

The Karnaphuli River estuary, located in southeast coast of Bangladesh, is largely exposed to heavy metal contamination as it receives a huge amount of untreated industrial effluents from the Chottagram City. This study aimed to assess the concentrations of five heavy metals (As, Pb, Cd, Cr and Cu) and their bioaccumulation status in six commercially important fishes, and also to evaluate the potential human health risk for local consumers. The hierarchy of the measured concentration level (mg/kg) of the metals was as follows: Pb (13.88) > Cu (12.10) > As (4.89) > Cr (3.36) > Cd (0.39). The Fulton’s condition factor denoted that fishes were in better ‘condition’ and most of the species were in positive allometric growth. The bioaccumulation factors (BAFs) of the contaminants observed in the species were in the following orders: Cu (1971.42) > As (1042.93) > Pb (913.66) > Cr (864.99) > Cd (252.03), and among the specimens, demersal fish, *Apocryptes bato* appeared to be the most bioaccumulative organism. Estimated daily intake (EDI), target hazard quotient (THQ), hazard index (HI) and carcinogenic risk (CR) assessed for potential human health risk implications suggest that the values were within the acceptable threshold for both adults and children. However, calculated CR values indicated that both age groups were not far from the risk, and HI values demonstrated that children were nearly 6 times more susceptible to non-carcinogenic and carcinogenic health effects than adults.

## Introduction

Heavy metals pollution has become a major concern worldwide due to their toxicity, intrinsic persistence, non-biodegradable nature, and accumulative behaviors [1]. These metals differ from other toxic materials in a way that they are neither created nor destroyed by human. They are inert in the environment and are often considered to be conservative pollutants if left undisturbed [2]. However, the rapid industrialization, urbanization, population growth, agricultural and other human activities have resulted in severe pollution by heavy metals globally, especially in developing countries [3]. Significant quantities of heavy metals from such activities are discharged into rivers, which can be strongly accumulated and biomagnified along water, sediment, and aquatic food chain, resulting in sublethal effects or death in local fish populations [4]. As fishes occupy higher trophic level in the food chain, they are considered one of the most common bioindicators for pollutants [5, 6]. Again, fishes are consumed by human as a major source of protein for many years. Thus, the human body is largely susceptible to enriched heavy metal concentration in fishes [7]. Consequently, an analysis of the levels of heavy metals in fish could be used to investigate anthropogenic impacts on ecosystem and human health.

Generally, bioaccumulation and biomagnification occur due to longstanding anthropogenic activities within a coastal ecosystem [8]. The accumulation of heavy metals in fish organs could also be driven by physiochemical and biological variables such as pH, temperature, hardness, exposure duration, feeding habits of species and habitat complexity [9]. While terrestrial species exhibit a strong pattern of biomagnification, marine and estuarine organisms show less clear pattern [10]. Condition factor, is one of the most common tools that is widely used to assess the life, reproduction and health conditions, as well as the life cycle of a fish species [11]. Along with that, condition factor also suggests the food availability and quality, breeding duration, and process for distinct populations [12, 13]. In addition, this tool indicates the status of fish health due to stress in the population within an ecosystem [14].

Heavy metals and metalloids, when occurring at higher concentrations, become severe poisons for all living organisms including human. For example, an excessive amount of Hg, As, Pb, and Cd elements could be detrimental to the living cells, and a prolonged exposure to the body can lead to illness or death [15]. Among the metals, Hg is the most toxic metal in our environment. Methyl mercury toxicity are inhibition of protein synthesis, microtubule disruption, increase of intracellular Ca^2+^ with disturbance of neurotransmitter function [16]. Prostatic proliferative lesions, lung cancer, bone fractures, kidney failures are associated with chronic exposure to Cd, even at a low concentration of ~ 1 mg/kg [17, 18]. Excessive Pb can have detrimental health effect on human [19] including nervous system disorders, mental retardation, skeletal hematopoietic function disorder and even death [20]. Cr was reported to have carcinogenic effects on human health [21, 22].

The Karnaphuli River estuary is one of the potential fish habitats along the southeast coast of Bay of Bengal known to be an important breeding, feeding and nursery ground for many aquatic species. At present, the ecosystem is receiving untreated effluents from several industries including textile crafts, dyeing industry and others as it passes through the industrial zone [23]. A number of studies have attempted to assess the contamination status from river and estuarine environment from Bangladesh [3, 24–27], from China [28], from Turkey [29]. However, to date there has been no proper investigation carried out on the potential human health risk due to heavy metal contamination in the fish species harvested and consumed from the Karnaphuli estuary. Therefore, this study aimed to fill this knowledge gap by assessing the concentration of heavy metals in some selected fish species and their bioaccumulation status, and the human health risk for local adult and children consumers.

## Materials and methods

### Ethical statement

Specimens from wild populations were collected from local fishermen. None of the sampled species were endangered or protected. No permit was required to conduct the present study. There were no ethical considerations linked to the experiment.

### Sampling

**Fig 1:**
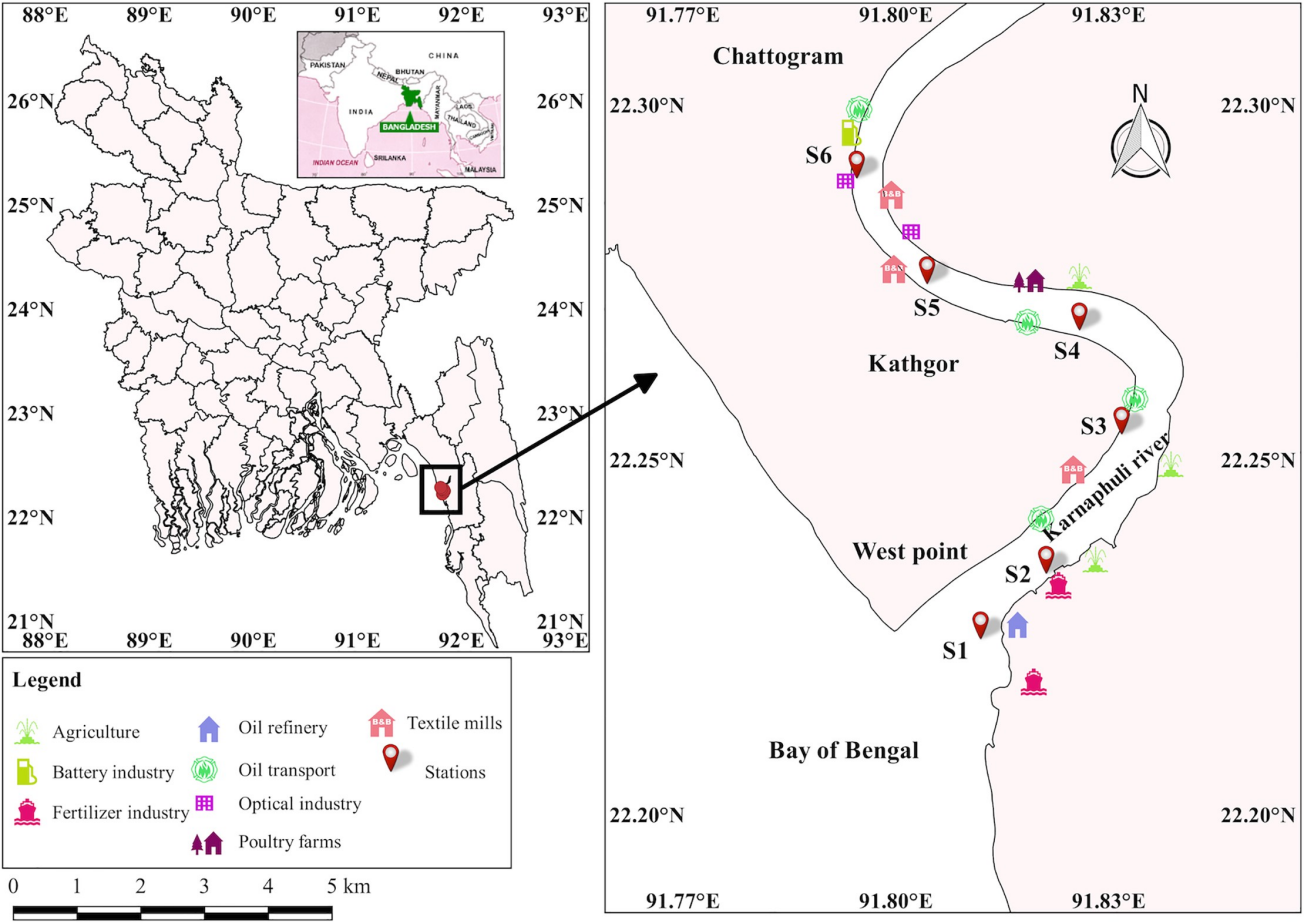
Six sampling locations in Karnaphuli River estuary and the source of heavy metal pollution. [Reprinted from (www.wikipedia.org) under a CC BY license, with permission from (Wikipedia), original copyright (2007)].

The study area (Karnaphuli River estuary) is located from 22.234008 N and 91.821105 E to 22.289695 N and 91.794403 E (Fig 1). A total of six commercial species (i.e. *Apocryptes bato* (Chewa), *Pampus chinensis* (Rup chanda), *Liza parsia* (Bata), *Mugil cephalus* (Flathead bata), *Hyporhamphus limbatus* (Ek Thuitta), *and Tenualosa toil* (Chandana ilish) were collected from fishermen for a period of seven months (February 2018 to August 2018) using seine net. The collected samples were carried in plastic ice container and immediately stored at –20°C. Afterwards in the lab, total length (cm) and weights (gm) were measured carefully to the nearest 0.1 cm using a vernier caliper; total weight was determined with an electronic balance to 0.01 g accuracy. Muscles of each specimen were dissected with stainless steel scissors for further chemical analysis using inductively coupled plasma mass spectrometry (ICP-MS, Model: ELAN9000, Perkin-Elmer, Germany) for metal detection. Data were analysed statistically by fitting a straight line adopting the least square method.

### Chemical analysis procedures and accuracy

A 1.5 g of dissected muscle was used for subsequent analysis. The samples were dried in a Zirbus freeze drying machine (Model: VaCo 2, Germany, CFC free, condenser dimensions-Ø200×200 mm) for 24 h [30]. After cooling, 0.5 g of muscle samples were placed in 10ml of of 100% HNO_3._ The solution was heated on an oil bath and added with 4 drops of H_2_O_2_ repeatedly until the mixture turned clear. The solution was mineralized using microwave digester (model: WX-6000, China). After filtering, solution was diluted for 2h and kept in a 50 ml tezaron tube [3]. A standard reagent (Merck VI; Germany) was used to analyze the metal concentration in the targeted fish species, which was prepared from the same acid matrix [1]. Moreover, certified reference material (CRM 320, Merck KGaA, Germany) was employed for validation and accuracy check [3].

### Metal pollution index (MPI)

To assess the metal pollution, the metal pollution index *(*MPI) was adopted following [31] and [32]. The equation is as follows:

$$MPI={\left({CM}_{1}\times {CM}_{2}\times {CM}_{3}\times \ldots ..\times {CM}_{n}\right)}^{{1/}_{n}}$$

where, CM_1_ is the concentration value of first concerned metal, CM_2_ is the concentration value of second concerned metal, CM_3_ is the concentration of third concerned metal, CM_n_ is the concentration of n_*th*_ metal (mg/kg dry wt) in the tissue sample of a certain species.

### Statistical analysis

The mean and standard deviation of metal concentrations were calculated. The Kolmogorov-Smirnov, Shapiro-Wilk and Kruskal-Wallis tests were performed using SPSS 23. The Kolmogorov-Smirnov and Shapiro-Wilk tests were performed to check the normality of the data [33]. The Kruskal-Wallis test was carried out to identify significant variance of the targeted elements in the specimens of the studied area where p ≤ 0.05 was used as the cut-off for significance (confidence level in 95%). In addition, the Levene’s test with the significant level at p ≤ 0.05 was adopted to determine the homogeneity of variances in terms of ANOVA tests. Correlation matrix (CM) and principal component analysis (PCA) were performed to determine the correlations and association between heavy metals in the studied fish species [34]. The correlation matrix used to analyze heavy metals could result in both positive and negative outcomes [35–38]. Hierarchical cluster analysis is one of the most widely used hierarchical algorithms which results in clusters in which variables or individuals are added in sequence considering hierarchy of the cluster [39]. Additionally, the similar groups of the studied elements within the sampling sites measured as a distance between the two closest members, and is conducted by Origin 9 software [3, 40]. Metals with similar properties were pooled in one or associated cluster while the dissimilar groups of elements were plotted in a separate cluster, and thus differentiated the contamination status of the samples [41–43].

### Bioaccumulation factor

Bioaccumulation factors (BAFs) were calculated as a ratio between the concentration level of biota (those in water) and the living environment of the specimens, and was expressed as follows [44–46]:

$$BAF=\frac{{Cn}_{Biota}}{{Cn}_{Water}}$$

where *Cn*_*Biota*_ is the concentration of metal in the tissues (mg/kg) and *Cn*_*Water*_ is the metal concentration in the aquatic environment (mg/l). BAF is categorized as follows: BAF < 1000: no probability of accumulation; 1000 < BAF < 5000: bioaccumulative; BAF > 5000: extremely bioaccumulative [47].

### Length-weight relationship and condition factor

The length-weight relationship of the fish samples were calculated using Fulton condition factor following the equation [48–51]:

Q=100×WL3

where W is the total body weight of fish (gm), L is the total length of fish (cm). Fulton`s Q is categorized as follows: Q = 1: Condition is poor, Q = 1.2: condition is moderate, Q ≥ 1.40: condition is proportionally good [52]. The equation can be expressed by the following formula [12, 53]:

$$W={aL}^{b}$$

The equation can be estimated using the least-square formula adopted with the logarithm form of the equation is shown as [54]:

logW=loga+blogL

where ‘a' is the calculated intercept of the regression line, and ‘b’ is the coefficient of that regression. The ‘b’ values signify the growth pattern of an organism which can be classified as follows: b < 3: negative allometric, b = 3: Isometric and b > 3: positive allometric [48, 55].

As Fulton’s Q is substantially correlated with the length-weight relationship, exponent ‘b’ acts an identical role of determining the well-being of the organisms [48]. The deviation of the condition, further, depends on the food availability and the divergence of reproductive organ development [56].

## Human health risk

### Estimated daily intake (EDI)

Estimated daily intake (EDI) was calculated by the following equation [22, 57, 58]:

$$EDI=\frac{\left(Cn\times IGr\right)}{Bwt}$$

where C*n* is the concentration level of metal in the selected fish tissues (mg/kg dry-wt); *IGr* is the acceptable ingestion rate, which is 55.5 g/day for adults and 52.5 g/day for children [59, 60]; *Bwt* is the body weight: 70 kg for adults and 15 kg for children [59].

### Target hazard quotient (THQ) for non-carcinogenic risk assessment

THQ was estimated by the ratio of EDI and oral reference dose (RfD). RfDs of the different metals for example As, Pb, Cd, Cr, and Cu are 0.0003, 0.002, 0.001, 0.003 and 0.3, respectively [59, 61]. The value of ratio < 1 implies a non-significant risk effects [62]. The THQ formula is expressed as follows [60, 63–65]:

$$THQs=\frac{E_{d}\times E_{p}\times EDI}{A_{t}\times RfD}\times 10^{-3}$$

Where *E*_*d*_ is exposure duration (65 years) (USEPA, 2008); *E*_*p*_ is exposure frequency (365 days/year) [21]; *A*_*t*_ is the average time for the non-carcinogenic element (*E*_*d*_×*E*_*p*_).

### Hazard index (HI)

Hazard index (HI) was calculated for the multiple elements (Hg, As, Mn, and Cr) found in the fish samples and the equation is as follows [3, 19, 66, 67]:

$$HI=\sum_{i=k}^{n}THQs$$

where, THQs is the estimated risk value for individual metal [68]. When HI value is higher than 10, the non-carcinogenic risk effect is considered high for exposed consumers [69–71].

### Carcinogenic risk (CR)

To assess the probability of developing cancer over a lifetime, the carcinogenic risk is evaluated for the consequence of exposure to the substantial carcinogens [72, 73]. The acceptable range of the risk limit is 10^−6^ to 10^−4^ [74–77]. CRs higher than 10^−4^ are likely to increase the probability of carcinogenic risk effect [78, 79]. The established equation to assess the CR is as follows [60, 61, 80, 81]:

$$CR=\frac{E_{d}\times E_{p}\times EDI\times CSf}{A_{t}}\times 10^{-3}$$

Where CSf is oral slope factor of particular carcinogen (mg/kg-day) [74]. Available CSf values (mg/kg-day) are: As (1.5), Pb (0.0085) and Cd (6.3) [74].

## Results

### The concentration of heavy metals and source identification

The average concentration of Pb, Cu, As, Cr, and Cd from the fish tissues were 13.88 mg/kg (range: 3.19–6.19 mg/kg); 12.10 mg/kg (range: 10.27–16.41 mg/kg); 4.89 mg/kg (range: 3.19–6.19 mg/kg); 3.36 mg/kg (range: 2.46–4.17 mg/kg), and 0.39 mg/kg (range: 0.21–0.74 mg/kg) respectively. Therefore, the hierarchy of the metal concentrations was: Pb > Cu > As > Cr > Cd (please see Table 1). From the results, Pb was 1.14, 2.8, 4.13, and 35.44 folds higher than Cu, As, Cr, and Cd respectively, and contributed 40% of all the elements in the study area. Along with that, Pb attributed a higher concentration of 15.73 mg/kg at S1, whereas Cd was accounted for lower concentration of 0.29 mg/kg on average observed at S1.

**Table 1:** Concentration of heavy metals of different species and their feeding nature, length and weight and a comparison of other relevant studies along with various standard guideline values.

Species	Feeding nature	Amounts	Length (cm)	Weight (gm)	Heavy metals (mean ± std) mg/kg						MPI	References
					As	Pb	Cd	Cr	Cu			
*A*. *bato*	Demersal	18	13.26±1.35	72.64±6.39	4.65±1.06	15.22±1.32	0.60±0.13	3.54±0.54	15.29±3.82	4.70	
*P*. *chinensis*	Demersal	18	6.96±0.90	166.58±21.68	5.03±0.86	14±1.79	0.44±0.14	3.59±0.55	13.10±2.49	4.29	
*L*. *parsia*	Demersal	18	7.34±1.17	25.20±3.12	4.36±0.93	13.98±1.93	0.34±0.09	3.30±0.40	9.50±1.16	3.65	
*M*. *cephalus*	Pelagic	18	27.17±2.30	711.44±111.03	4.89±0.48	12.70±1.72	0.31±0.09	3.14±0.36	11.48±1.27	3.69	
*H*. *limbatus*	Pelagic	18	10.97±0.80	26.77±4.39	5.14±0.86	13.77±1.54	0.35±0.08	3.52±0.48	12.52±1.40	4.05	
*T*. *toli*	Pelagic	18	30.68±2.54	646.82±41.16	5.26±0.49	13.61±0.82	0.31±0.07	3.11±0.54	10.72±1.47	3.75	
*Water (mg/l)*					0.006±0.003	0.017±0.006	0.002±0.001	0.006±0.002	0.006±0.001		
**Guidelines (mg/kg)**											
FAO					1	2.5	0.2	1	10		[82]
WHO					0.01	2	–	0.15	3		[83]
EU					–	0.1	0.05	1	3		[84]
Bangladesh (fish)					5	0.30	0.25	–	5.00		[85]
**Literature (mg/kg)**											
Coastal area, Bangladesh					0.08–13	0.07–0.63	0.03–0.09	0.15–2.2	1.3–14		[86]
Arasalar River, India					–	0.23	6.13	0.3	–		[87]
North east coast, India					0.64	–	0.33	–	3.9		[88]
Ganga River, India					–	3–6	0.1–2.9	–	10–100		[89]
Pearl River, China					–	8.64	8.55	8.73	2.48		[90]
Meiliang Bay, China					–	0.636	0.173	0.118	0.336		[91]
Iskenderun Bay, Turkey					–	0.09–6.95	0.01–4.16	0.07–6.46	0.04–5.43		[92]

The evaluated MPIs ranged from 3.65 mg/kg to 4.70 mg/kg with the mean of 4.02 mg/kg (Table 1). Due to higher concentration level, the maximum MPI value (4.70 mg/kg) was corresponded to *A*. *bato*, followed by *P*. *chinensis* (4.2 mg/kg) *H*. *limbatus* (4.05 mg/kg), *T*. *toli* (3.75 mg/kg), *M*. *cephalus* (3.69 mg/kg), and *L*. *parsia* (3.65 mg/kg).

From the statistical results, Kolmogorov-Smirnov and Shapiro-Walk test revealed that the metals in the targeted fish species were non-normally distributed along the study area. The adopted Levene’s tests showed that metals were non-homogenously distributed. Kruskal-Wallis test identified that the distribution of metal was significantly different (p ≤ 0.05) in the fish species along the sampling stations. From Table 2, among the species, *L*. *parsia and H*. *limbatus* exhibited significant relationship (regression line) with As only, where *T*. *toil* showed significant relationship with As and Pb (p ≤ 0.05). None of the other metals showed a significant linear relationship with the organisms. Among the species, *T*. *toil* showed the maximum response for Pb (R^2^ = 99.5%), followed by *L*. *parsia* for As (R^2^ = 83.7%).

**Table 2:** A regression analysis between metal distributions in the selected specimens.

Species and metals	Regression equation	Standard error	Pearson’s r	P values	R^2^ (%)
*A*. *bato*					
As	y = 8.66+0.99x	0.404	0.774	0.070	58.1
Pb	y = 11.88+0.09x	0.511	0.088	0.867	0.8
Cd	y = 13.44–0.31x	5.166	-0.029	0.955	0.01
Cr	y = 12.70+0.16x	1.25	0.063	0.904	0.4
Cu	y = 11.56+0.11x	0.168	0.313	0.545	9.8
*P*. *chinensis*					
As	y = 4.19 + 0.55x	0.449	0.522	0.287	27.3
Pb	y = 5.14 + 0.13x	0.243	0.257	0.621	6.6
Cd	y = 5.72 + 2.81x	3	0.425	0.4	18.1
Cr	y = 5.75 + 0.34x	0.8	0.205	0.695	4.2
Cu	y = 4.58 + 0.18x	0.157	0.502	0.309	25.3
*L*. *parsia*					
As	y = -0.96 + 0.73x	0.16	0.914	**0.01**	83.7
Pb	y = 7.79 + 0.84x	0.711	0.51	0.301	26.0
Cd	y = 0.12 + 0.03x	0.036	0.378	0.459	14.3
Cr	y = 1.86 + 0.2x	0.139	0.58	0.227	33.7
Cu	y = 4.24 + 0.72x	0.346	0.719	0.107	51.7
*M*. *cephalus*					
As	y = 2.08+0.11x	0.09	0.5	0.311	25.0
Pb	y = 23.98–0.42x	0.309	-0.557	-0.557	31.0
Cd	y = 0.87–0.02x	0.016	-0.547	0.26	30.0
Cr	y = 4.04–0.03x	0.075	-0.217	0.68	4.7
Cu	y = 16.95–0.21x	0.257	-0.364	0.477	13.3
*H*. *limbatus*					
As	y = -4.63+0.89x	0.309	0.821	**0.045**	67.5
Pb	y = 1.09+1.156x	0.778	0.596	0.211	35.6
Cd	y = 0.36–0.001x	0.048	-0.014	0.978	0.01
Cr	y = -0.6+0.38x	0.238	0.620	0.189	38.5
Cu	y = 2.54+0.91x	0.749	0.519	0.291	26.9
*T*. *toli*					
As	y = 0.48+ 0.16x	0.059	0.8	**0.05**	63.9
Pb	y = 3.74+0.32x	0.012	0.997	**1.11E-5**	99.5
Cd	y = 0.35–0.01x	0.013	-0.042	0.936	0.2
Cr	y = -1.22+ 0.14x	0.08	0.662	0.152	43.8
Cu	y = 0.37 + 0.34x	0.237	0.581	0.227	33.7

**Fig 2:**
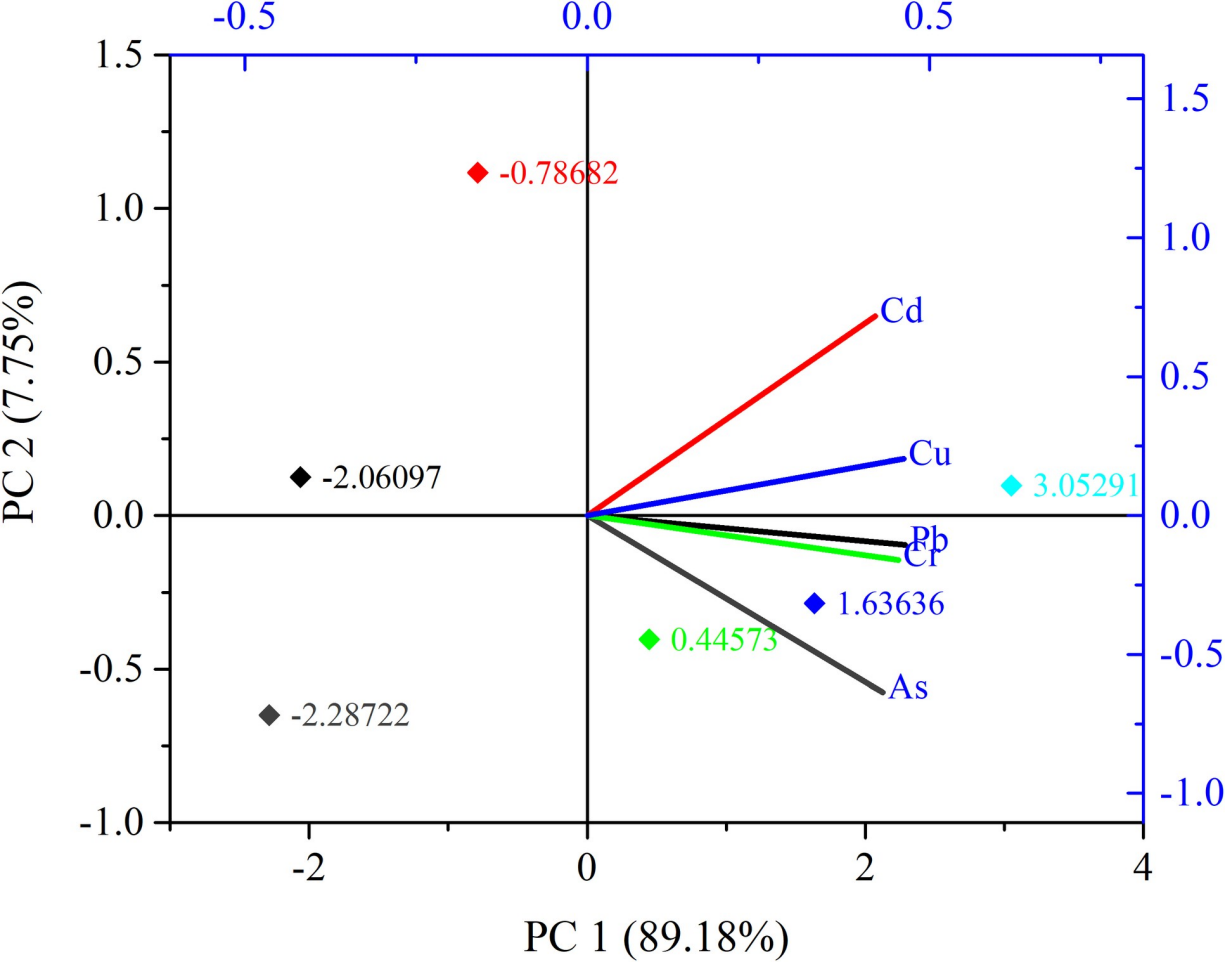
Loading plots of principal component analysis (PCA) of elements.

Table 3 depicts the correlation matrix among the metals in different fish species. The most significant positive correlations were observed between As—Pb (r = 0.912), As–Cr (r = 0.904), Pb–Cr (r = 0.917), Pb–Cu (r = 0.967), and Cd–Cu (r = 0.911) at p <0.05 level. Based on CM, the rotated component plot, PCA was depicted in Fig 2. The PCA exhibited two factors PC 1 and PC 2, which resulted in the corresponded variance of 89.18% and 7.75% respectively. Both factors were responsible for cumulative variance of 96.93%. Cu and Pb were the most dominant elements in PC 1, corresponded with the loadings of 20.78% and 20.70% respectively. On the contrary, in PC 2, Cd and Cu showed the domination with the loadings of 77.81% and 22.19% respectively.

**Table 3:** Pearson’s correlation coefficient between heavy metal elements and matrix of PCA loadings.

Correlation analysis						Principal component analysis	
	As	Pb	Cd	Cr	Cu	PC 1	PC 2
As	1					0.432	-0.637
Pb	0.912*	1				0.465	-0.106
Cd	0.640	0.828	1			0.421	0.718
Cr	0.904*	0.917*	0.820	1		0.455	-0.160
Cu	0.840	0.967*	0.911*	0.890	1	0.463	0.205
Eigenvalues						4.459	0.387
% of Variance						89.18	7.75
Cumulative %						89.18	96.93

* Significant at p ≤ 0.05

**Fig 3:**
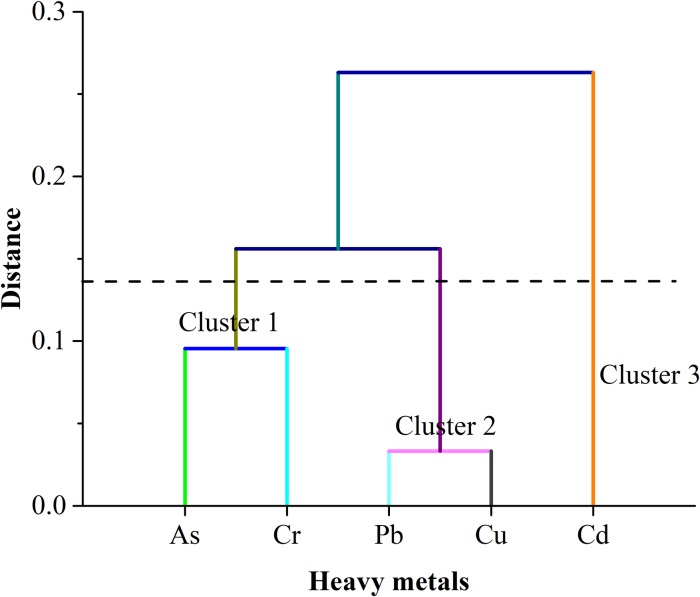
Hierarchical cluster (dendrogram) using Ward linkage method among the experimented metals in fish species.

To execute hierarchical cluster dendrogram, the Ward-Linkage method was employed with Euclidean distance, which resulted in three distinct clusters, presented in Fig 3. Cluster 1 included As and Cr that could have been originated from anthropogenic activities like chemical industries. Pb, and Cu confined in cluster 2 could have been attributed from the textile organization, fertilization and oil droppings from the boats/ships in the study area. Lastly, Cd was included in cluster 3, and source of the Cd could have come from battery industry.

### Length-weight Relationship and condition factor evaluation

Higher ‘b’ value reflected the appetite state and reproductive organ development of the species [55]. The identified b value of *L*. *parsia* from the length weight relationship was close to 3, hence, it represented isometric growth pattern that was considered as ideal shape (Table 4). Meanwhile, among all, species, *P*. *chinensis* exhibited the highest positive allometric growth, which was 37 times higher, than average value and 14 folds, on average, higher than other species.

**Table 4:** Length-weight relationship, growth pattern and Fulton condition factor for the targeted fishes.

Species	a (intercept) ± SE	b (slope regression) ± SE	Group (Growth pattern)	W = aL^b^	Fulton`s Q ± std	Fish Condition
*A*. *bato*	19.62 ±16.63	3.99±1.24	Positive allometric	W = 19.62×L^3.99^	3.22±0.78	Good
*P*. *chinensis*	7.23±25.94	22.88±3.7	Positive allometric	W = 7.23×L^22.88^	51.77±15.26	Good
*L*. *parsia*	7.13±3.8	2.76±0.51	Isometric	W = 7.13×L^2.46^	7.10±3.44	Good
*M*. *cephalus*	0.002±0.0008	10.85± 4.98	Positive allometric	W = 0.002×L^10.85^	3.56±0.45	Good
*H*. *limbatus*	0.01±0.002	2.43±0.41	Negative	W = 0.01×L^2.43^	2.03±0.23	Good
*T*. *toli*	164.30±60.97	15.72±1.98	Positive allometric	W = 164×L^15.72^	2.29±0.46	Good

### Bioaccumulation (BAF) status of targeted species

**Fig 4:**
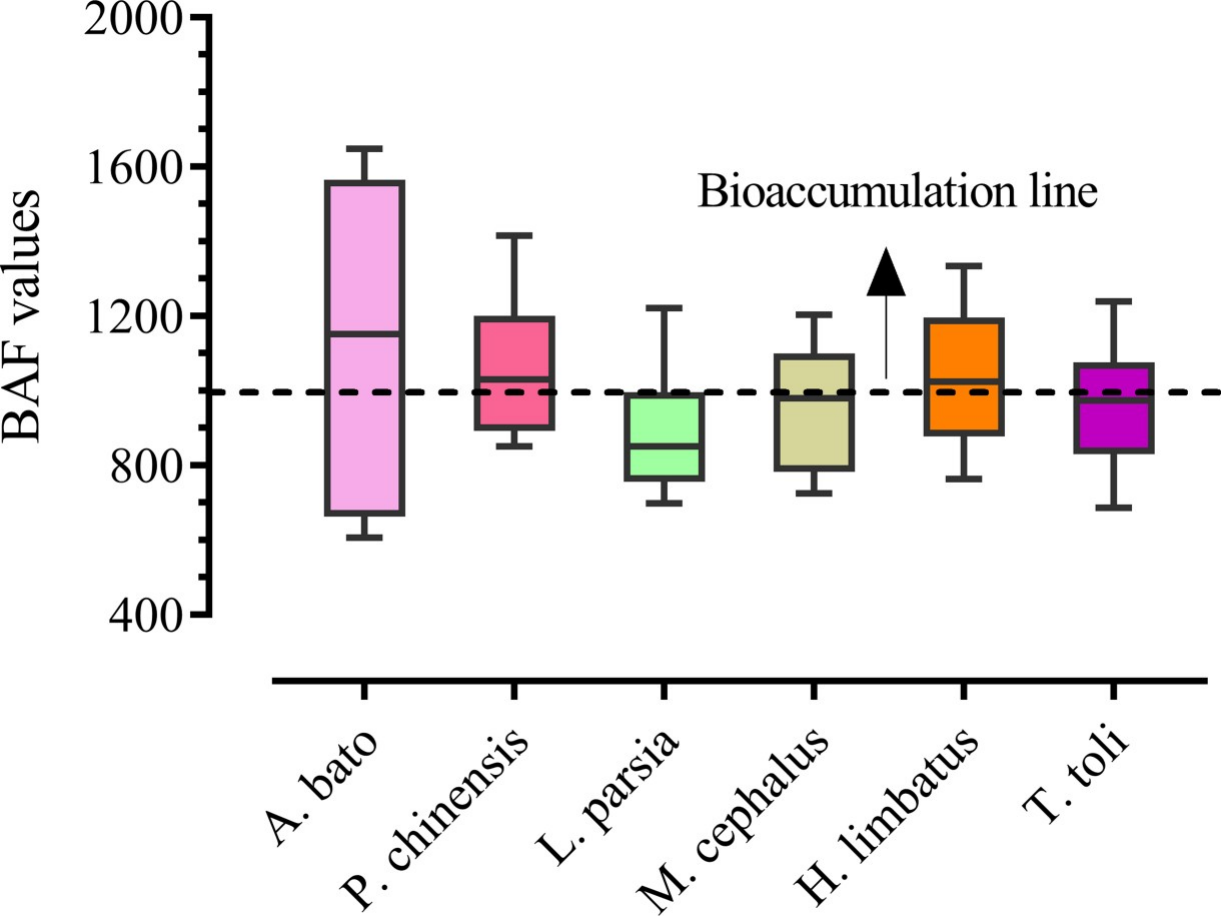
Bioaccumulation factor among the species that varied from particular metals and species.

The estimated BAFs are depicted in Fig 4. The BAFs were ranged from 110.53 for Cd observed in S4 to 3353.7 for Cu as well. The minimum value was found for *T*. *toil*, on the other hand, *A*. *bato* showed maximum bioaccumulation result. Moreover, the mean BAFs of the metals were observed in the species as follows: Cu (1971.42) > As (1042.93) > Pb (913.66) > Cr (864.99) > Cd (252.03).

## Health risk evaluation

### Estimated daily intake (EDI)

The explored EDI of two concerned age groups, adults and children, is presented and summarized in Table 5. Adults and children showed comparably higher EDIs for demersal species than pelagic ones. In the consequence, high doses of demersal species were exposed to the consumers through consuming metal affected fish species as the food items. The EDIs for both groups were organized in the following order: Pb > Cu > As > Cr > Cd.

**Table 5:** A comparison between recommended daily allowance (RDA) and estimated daily intake (EDI) for adults and children.

Elements	Mean concentration (mg/kg)	RDA (mg/kg/person) [93]	EDIs (mg/day/person)	
			Adult	Child
As	4.89	0.15	0.005	0.029
Pb	13.88	0.25	0.011	0.049
Cd	0.39	0.07	0.001	0.001
Cr	3.36	0.23	0.003	0.012
Cu	12.10	35	0.010	0.042

### Target hazard quotient (THQ) and Hazard Index (HI) for non-carcinogenic risk

The assessed target hazard quotient (THQ) for the studied fish species are displayed in Table 6. THQs in the adult group induced to As, Pb, Cd, Cr, and Cu were 0.016, 0.003, 3.0E-04, 0.001 and 2.38E-04, respectively, whereas for children the values were 0.097, 0.014, 0.001, 0.004 and 0.001, respectively. Moreover, the rank of the THQs of the elements was as follows: As > Pb > Cr > Cd > Cu. While, for the cumulative scenario of HI, children were 5.83 times more susceptible than adults. However, the investigated HI was not surpass the recommended limit (Table 6).

**Table 6:** Calculated THQ, HI and CR for the selected two aged groups.

Species	THQ (As)		THQ (Pb)		THQ (Cd)		THQ (Cr)		THQ (Cu)		HI		CR (As)		CR (Pb)		CR (Cd)	
	Ad	Ch	Ad	Ch	Ad	Ch	Ad	Ch	Ad	Ch	Ad	Ch	Ad	Ch	Ad	Ch	Ad	Ch
*A*. *bato*	0.012	0.054	0.003	0.015	0.000	0.002	0.001	0.004	0.000	0.001	0.017	0.077	5.47E-06	2.44E-05	1.79E-05	8.00E-05	2.99E-06	1.30E-05
*P*. *chinensis*	0.013	0.059	0.003	0.014	0.000	0.002	0.001	0.004	0.000	0.001	0.018	0.08	5.93E-06	2.64E-05	9.35E-08	7.40E-05	2.18E-06	9.70E-06
*L*. *parsia*	0.011	0.051	0.003	0.014	0.000	0.001	0.001	0.004	0.000	0.001	0.016	0.071	5.14E-06	2.29E-05	9.33E-08	4.20E-07	1.68E-06	7.50E-06
*M*. *cephalus*	0.013	0.057	0.003	0.013	0.000	0.001	0.001	0.004	0.000	0.001	0.017	0.076	5.77E-06	2.57E-05	8.48E-08	3.80E-07	1.52E-06	6.80E-06
*H*. *limbatus*	0.013	0.06	0.003	0.014	0.000	0.001	0.001	0.004	0.000	0.001	0.018	0.08	6.06E-06	2.7E-05	9.2E-08	4.10E-07	1.72E-06	7.70E-06
*T*. *toli*	0.030	0.3	0.003	0.014	0.000	0.001	0.001	0.004	0.000	0.001	0.034	0.319	1.35E-05	1.35E-04	9.09E-08	4.00E-07	1.54E-06	6.90E-06
Mean	0.016	0.097	0.003	0.014	0.000	0.001	0.001	0.004	0.000	0.001	0.015	0.079	6.98E-06	4.36E-05	3.07E-06	2.58E-05	1.94E-06	8.64E-06

### Carcinogenic risk (CR)

Exposure of CR was estimated for a particular element and summarized in Table 6. The measured CR values of As, Pb and Cd were ranged from 5.14E-06- 1.35E-05, 8.48E-08- 1.79E-05 and 1.52E-06- 2.99E-06 respectively in adults and 2.29E-05- 1.35E-04, 3.78E-07- 7.99E-05 and 6.76E-06- 1.33E-05 in children. The results showed that children were exposed to higher CRs than adults. But, calculated CR values for both age groups were noted far from the risk as acceptable range is 10^−6^ to 10^−4^.

## Discussion

### Concentration of heavy metals and source identification

In the present study, *T*. *toil* had the highest concentration of As, whereas *A*. *bato* exhibited the maximum concentration for Pb, Cd and Cu, and *P*. *chinensis* had the highest concentration of Cr. The metal concentrations in the edible tissues were ranked in the following sequence: *A*. *bato > P*. *chinensis > H*. *limbatus > T*. *toli > M*. *cephalus > L*. *parsia*. Data from the previous literatures showed that metal concentrations in fish muscles varied widely depending on the location and species. The As and Pb concentrations in this study were higher than all other findings and recommended guidelines. Although Cd concentration was at the lower level comparing Northeast coast, Ganga River, and Pearl River, it surpassed all the guideline values along with other coastal environments, Meiliang Bay, Iskenderun Bay, Arasalar River and coastal area of Bangladesh. The results reported by [89] from the Ganga River were generally lower than present results, except Cu. The concentrations of Cd and Cu in the fish collected from the Pearl River were higher than present study. Different fish species of Bangladesh’s coastal area have been reported to contain 0.08–13 As, 0.07–0.63 Pb, 0.03–0.09 Cd, 0.15–2.2 Cr and 1.3–14 Cu mg/kg dry weight of muscles [86], which were generally lower than the present findings. In this study, the demersal species had comparatively higher concentration level of metals than pelagic ones as they inhabit close to the bottom or sediment. The MPI values of 3.65 to 4.70 in the present study were much lower than those of Blackchin tilapia, *Sarotherodon melanotheron* and Silver catfish, *Chrysichthys nigrodigitatus* (8.1 to 17.76) from Okrika Estuary, Nigeria. This is most likely due to the oil bunkering and transportation activities along the study sites [94]. The findings of MPI in the present study were almost similar to that of *Rutilus rutilus* in Pluszne Lake [12]. However, the metal accumulation in fishes could be highly influenced by sampling locations and habitats [95, 96].

Previous studies have shown that heavy metals in the aquatic environment could come from different natural and anthropogenic sources [1, 3, 39, 97], where many factors influence their concentrations e.g., the original levels of rocks and parent materials, processes of soil formation, contamination by human activities, and other anthropogenic factors [98]. Generally, high correlations between specific heavy metals in the environment may reflect similar levels of contamination and/or release from the same sources of pollution [40]. In this study, strong positive correlations were found between Cd and Pb, Cr and Pb, Cr and Cd, and Cr and Cu indicating that they had the same source either natural or anthropogenic. The employed PCA revealed that the source of origin of the metal was anthropogenic. Pb, Cd, and Cu were the dominant compounds in PCA analysis due to their high loading scores. PCA also revealed the changes of geochemical composition and the justification of cluster analysis [99]. In the cluster analysis, Pb, Cd and Cu are grouped together in cluster 2, indicating that the anthropogenic sources of these heavy metals are closely related to the sediments of the study area. There are several manufacturing industries near the study area which largely use alloy, paints and poisonous chemicals containing As. The commercial uses of heavy metals in modern microelectronic and optical industries are notable sources of As intrusion in the aquatic environment [100]. Non-essential element, Pb originated from extreme agriculture, poultry farms, industries and textile mills might end up in the aquatic ecosystem [101], thus contributing to the metal in the study area. Thus the benthic feeders are to be greatly affected by the deposited Pb in the ecosystem [91]. Cd is typically found at a low concentration in the aquatic environment, however, indiscriminate use of phosphate fertilizer and industries are two primary sources of Cd introduction [102]. In the study area, nickel-cadmium battery manufacturing along with industries engaged with Cd metal incineration and production might increase the Cd concentration level in the aquatic environment [103, 104]. Besides Cd and Pb, Cr is also widely introduced in the textile industries [105]. Near the bank of the Karnaphuli estuary, such commercial textile industries produce colour pigment and thus become a common contaminant for the aquatic ecosystem [106]. Notably, a considerable level of Cu become swelled up in the study area due to oil droppings from ships and boats, recurrent usage of antifouling paints and other boating interferes [107].

### Bioaccumulation (BAF) status of target species

The bioaccumulation potential of metals was assessed in the muscles of various fish species, and was found to vary from species to species. The hierarchy suggested that most of the species were tend to be bioaccumulative (BAF value close to 1000). *A*. *bato* exhibited the highest bioaccumulation in the studied area. The accumulation of the metal elements in an aquatic organism depends upon the classification of species, invasion pathways, metabolic characters of the sampled tissues and finally, the surrounding environmental condition in which the species live [108]. In our results, the BAFs of As, Cd, Cr, Pb and Cu were relatively higher than those of Pearl River estuary [90], where the BAFs in Tilapia were reported to be Cd > Cu > Pb > Cr. Such reports were mostly in line with our results. The fact is that, Cu is actively persistent in muscles due to being an essential element of living tissue [60, 109]. Notably, the bioaccumulation capability of Cd takes a long time to spare, thus making it relatively infirm [20].

### Human health risk evaluation

EDI, based on the oral reference dose (RfD) for an individual element [110], reflects the daily exposure to the toxic element, and is executed to avoid any harmful effect on human health [63]. The records of EDI of the people were compared with recommended daily allowance (RDA), provided by WHO, and introduced that, mean EDI values of the metals were still lower than RDAs. The values lower than RDA guidelines suggested a lower possible health effect of the elements to the consumers. However, it would not be wise to take it as a permanent measurement to reach a final conclusion [61, 63].

The <1 THQ values for both adult and children suggested that the adverse effect on human health might not occur. Similarly, the HI results also followed the THQ trend. Hence, there is no potential non-carcinogenic effect for the consumers due to intake of the fish species. Studies carried out by several authors in similar conditions were in line with our results [32, 69, 111–113]. In general, the assessment of THQ for human health risk evaluation has no dose-response relation of the examined elements [114]. However, human can dramatically suffer in the long run due to multiple simultaneous pollutants [77].

The CR values lower than 10^−6^ indicates a negligible health risk whereas values in the range of 10−6–10^−4^ are in the acceptable belt [63]. The CR values found in this study suggested an acceptable limit, and consumers are therefore less prone to carcinogenic risk. In fact, 90% of the carcinogenic risk is observed in the As contaminated aquatic food items. The inorganic state of As is more lethal than organic one [20, 115], and only 10% of the total As can be assessed as inorganic form [63]. The findings of the present study were in the acceptable range (10^−6^ to 10^−4^) compared with Okogwu et al. [116], except for Cr which surpassed the CR limits. Again, in the Persian Gulf, consumers at the threshold limit for As were in concern of carcinogenic risk [117]. For this reason, carcinogenic risk should be given more attention due to intake of aquatic products, especially for the study area.

## Conclusions

Five heavy metals in the muscles of six fish species from the Karnaphuli River estuary were measured to investigate their potential sources, bioaccumulation rate and human health risk. Relatively high concentration of Cu was observed in *A*. *bato*. In most cases, the metal concentrations exceeded the recommended limits. The maximum metal accumulation was recorded in *A*. *bato*. *A*. *bato*, *P*. *chinensis* and *H*. *limbatus* were observed to be extreme bio-accumulative species. The mean bioaccumulation factors of the metals were observed in the studied species as follows: Cu > As > Pb > C > Cd.). Among the fishes studied, demersal fishes had higher levels of heavy metals compared with those fishes in the upper layers, suggesting higher level of heavy metals in the bottom water or sediment compared with the surface water. Accumulation of heavy metals in fish could be resulted from surface contact with the water, by breathing, and via the food chain. Heavy metals in the sediment enter the food chain through the feeding of benthic animals. Pearson correlation analysis showed strong positive correlations between Cd and Pb, Cr and Pb, Cr and Cd, and Cr and Cu indicating that they had the same source, either natural or anthropogenic. As per cluster analysis, Pb, Cd, and Cu, were grouped together, indicating the local anthropogenic sources. Existing textile mills, fertilizer factory, leather industry and agricultural activities in the catchment area of the river might be the sources of high concentration of Pb and Cd. Carcinogenic risk assessment suggested local consumers were free from the risk of cancer for the time being but they might be affected in future upon consumption of fish from studied region. Finally, it is found that children were six times more vulnerable to non-carcinogenic and carcinogenic risks than adults. Nonetheless, further study required ensuring the same conclusions are reached.
